# Silver Nanoclusters Decrease Bacterial Resistance to Heavy Metals and Antibiotics

**DOI:** 10.3390/nano16010054

**Published:** 2025-12-31

**Authors:** Gennady L. Burygin, Daniil S. Chumakov, Anastasia S. Astankova, Yulia A. Filip’echeva, Julia A. Balabanova, Yelena V. Kryuchkova

**Affiliations:** 1 Institute of Biochemistry and Physiology of Plants and Microorganisms, Saratov Scientific Centre of the Russian Academy of Sciences, 13 Prospekt Entuziastov, 410049 Saratov, Russia; laik2012@yandex.ru (D.S.C.); asastankova@gmail.com (A.S.A.); ljuche@yandex.ru (Y.A.F.); yulia-kusmarceva@yandex.ru (J.A.B.); kryu-lena@yandex.ru (Y.V.K.); 2 Department Organic and Bioorganic Chemistry, Institute of Chemistry, Saratov State University, 83 Astrakhanskaya Street, 410012 Saratov, Russia

**Keywords:** silver nanocluster, multidrug resistance, heavy metals, antibiotics

## Abstract

Nanomaterials are widely used in biomedical research as drug and antibody carriers, and some nanomaterials have been shown to exhibit antimicrobial activity. Previously, silver nanoclusters (AgNCs) were predicted to interact with the bacterial TolC protein, which is involved in the development of multidrug resistance in pathogens. In this study, glutathione-coated AgNCs were synthesized and characterized. Their toxicological properties were studied in a microplate assay against five bacterial strains, both as single components and in mixtures with heavy metal salts and antibiotics. The resulting AgNCs had a diameter of 2.2 ± 0.5 nm, with excitation and emission maxima of λ = 490 nm and λ = 638 nm, respectively. No significant growth inhibition was observed at the concentrations used in resistance modulation assays (≤2.5 µg/mL Ag), except for transient effects at very high concentrations. A decrease in bacterial resistance to copper (II) and cadmium (II) cations and the antibiotics erythromycin and levofloxacin was observed upon the addition of AgNCs containing 2.5 μg/mL silver to the nutrient medium. A dose-dependent effect of AgNCs on bacterial resistance to toxicants was established. Thus, nanoclusters can be considered as inhibitors of bacterial resistance to heavy metals and antibiotics, which may be useful in studying bacterial adaptation mechanisms and developing technologies for overcoming multidrug resistance in bacteria.

## 1. Introduction

Modern technology is capable of producing nanomaterials of various structures, compositions, and shapes [1,2], which are already finding active application in various fields [3,4], and the prospects for the introduction of nanomaterials in the near future are even more impressive [5]. One of the first applications of metal nanoparticles (NPs) was their use in biological and medical research as carriers of antibodies (e.g., for transmission electron microscopy (TEM) and immunoassays) and other biologically active molecules [6,7]. In the modern world, the use of nanomaterials in medicine also remains dominant and continues to develop.

One area of nanomaterial application is antimicrobial therapy [8] as antibiotic carriers, thanks to the direct antimicrobial properties of some nanomaterials. This area is especially relevant given that the rapid rise in antibiotic resistance is a critical global health threat, urging the exploration of innovative solutions to combat microbial infections. NPs have garnered significant attention for their antimicrobial properties, with potential applications across both the biomedical and environmental sectors [9]. Among many nanomaterials, nanoclusters show good promise for use in antimicrobial therapy [10,11].

Nanoclusters are supramolecular complexes measuring 1–4 nm and consisting of metal atoms and stabilizing organic molecules. Many nanoclusters exhibit luminescent properties. Their unique physical and chemical properties are based on a core–shell structure. The nanocluster core consists of a countable number of metal atoms. The ligand shell, on the one hand, influences the electronic structure of nanoclusters and their fluorescent properties, and on the other hand, ensures their stability, protecting the metal core from oxidative etching and aggregation [12,13,14,15,16,17]. The presence of a stable shell on the surface of silver nanoclusters (AgNCs) leads to their antimicrobial activity being significantly reduced relative to silver nanoparticles or silver ions (Ag^+^). In a review by Zheng et al. (2022) [11] summarized all data on the antimicrobial activity of metal nanoclusters and demonstrated that the primary mechanism of action of AgNCs on bacterial cells is the generation of Ag^+^. A number of studies have found that functionalization of AgNCs with various components (fibrils, DNA, nanoparticles, antibiotics, etc.) leads to disruption of the ligand shell stability and the release of Ag^+^, which consequently leads to a significant increase in antimicrobial activity [18,19,20,21]. The mechanisms of antibacterial activity of the AgNCs themselves (without the production of Ag^+^) are significantly less well understood. Some studies [10,22] have shown that nanoclusters are active at concentrations above 20 μg/mL towards Gram-negative bacteria (*Escherichia coli* and *Pseudomonas aeruginosa*) and above 50 μg/mL towards Gram-positive bacteria (*Staphylococcus aureus*). In the work of Jin et al. (2017) [10], it was suggested that AgNCs can block the functioning of porins in the outer membranes (for *Escherichia coli* strain DH5α) and the respiratory chain into cells (for *Escherichia coli* strain DSM 4230). Tumskiy et al. (2023) [23] predicted, based on molecular docking, the interaction of silver nanoclusters (AgNCs) with a diameter of 2 nm with a trimer of the TolC protein, which forms a channel in the outer membrane of gram-negative bacteria.

TolC proteins are components of various resistance-nodulation-cell division (RND) efflux pumps [24], which ensure the removal of various toxin compounds from cells: antibiotics, detergents, and heavy metal cations [25]. Accordingly, disruption of the functioning of efflux pumps upon interaction of nanoclusters with the TolC protein should lead to a decrease in the resistance of bacteria to toxicants by reducing or stopping the efflux. In our preliminary work [26], we found that the addition of AgNCs to the culture medium of the bacterium *Achromobacter insolitus* LCu2 reduces the resistance of the strain to copper cations. The effect of nanoclusters on resistance has not been studied for other bacteria and toxicants. In this regard, the aim of this work was to determine the effect of the presence of AgNCs in the culture medium of bacteria on resistance to heavy metals and antibiotics.

## 2. Materials and Methods

### 2.1. Synthesis of Silver Nanoclusters

**Figure 1:**
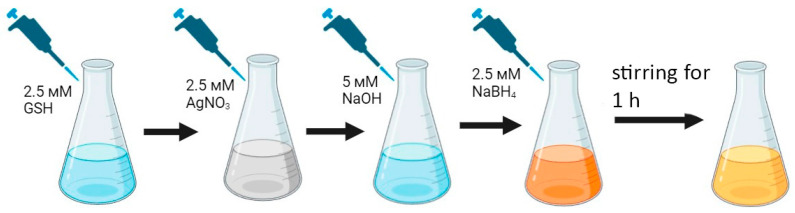
Scheme for the synthesis of glutathione-stabilized silver nanoclusters (GSH-AgNCs). The change in the color of the solution at each stage of GSH-AgNCs synthesis is schematically shown in different colors.

To obtain bioorganic metal nanoclusters, the same molecule is often used as a reducing agent and stabilizer. However, the synthesis of silver nanoclusters (AgNCs) stabilized by peptide ligands has its own characteristics [27]. To obtain fluorescent silver nanoclusters based on glutathione (GSH-AgNCs), we used sodium borohydride (NaBH_4_) as a reducing agent for the reduction of Ag^+^ to Ag^0^ and L-glutathione (GSH) as an organic stabilizing matrix. The synthesis of GSH-AgNCs was carried out according to the method described by Tumskiy et al. (2023) [23] with minor modifications. The synthesis scheme is shown in Figure 1. In the first step, 8 mL of milliQ H_2_O, 1 mL of 25 mM reduced GSH, and 1 mL of 25 mM silver nitrate (AgNO_3_) were mixed in a glass vial. The solution was stirred at 500 rpm at room temperature. A white precipitate formed, representing insoluble silver coordination compounds (GSH-Ag(I)). In the next step, 50 μL of 1 M NaOH were added to the vial, after which the solution became colorless. After 30 s, 96 μL of 260 mM freshly prepared sodium borohydride (NaBH_4_) were added dropwise to the reaction mixture with vigorous stirring. The solution was then stirred for 1 h at room temperature. The resulting nanocluster solution was yellow. The synthesized nanoclusters were incubated for 12 h at 4 °C. The resulting GSH-AgNCs were then washed from the dispersion medium using centrifugal ultrafilters (Corning Inc., Corning, NY, USA) with a cutoff of 5 kDa. The nanoclusters, washed from the dispersion medium, were redissolved in water.

### 2.2. Characteristics of Silver Nanoclusters

Several methods were used to conduct the physicochemical characterization of silver nanoclusters. Electron microscopic characterization of GSH-AgNCs was performed using a Libra 120 transmission electron microscope (TEM) (Carl Zeiss, Oberkochen, Germany). Samples were applied to nickel grids with a formvar substrate and then dried. Electron microscopic measurements were performed at an accelerating voltage of 120 kV. The extinction spectra of GSH-AgNCs were recorded using a Specord S-600 spectrophotometer (Analytik Jena, Jena, Germany). The fluorescence excitation and emission spectra of GSH-AgNCs were recorded using a Cary Eclipse spectrofluorimeter (Agilent Technologies Inc., Santa Clara, CA, USA). Fluorescence measurements were performed with a slit width of 20 nm. Measurements of the zeta potential and hydrodynamic radius of nanoclusters by dynamic light scattering (DLS) were performed using a Zetasizer Nano nanoparticle characterization system (Malvern Instruments, Malvern, UK). To determine the dependence of the zeta potential of particles on the pH of the medium, a solution of nanoclusters was added in a ratio of 1:10 to a solution of 0.1 M phosphate buffer with pH values of 3.0, 4.5, 6.0, 7.5, and 9.0. At least 10 measurements were carried out in triplicate. The stability of GSH-AgNCs in culture medium was assessed after the purification from the original dispersion medium using centrifugal filters with a 5 kDa molecular weight cut-off. The resulting pellet was redispersed in liquid LB medium for fluorescent measurements with a slit width of 20 nm.

### 2.3. Bacterial Strains and Culture Conditions

The bacterial strains *Escherichia coli* K12 (IBPPM 204; DSM 4230), *Staphylococcus aureus* ATCC 25923, *Pseudomonas aeruginosa* ATCC 9027, *Achromobacter insolitus* LCu2 (RCAM 04723; IBPPM 631) [28], and *Enterobacter cloacae* K7 (IBPPM 476) [29] were used in the work. Bacterial cultures were grown at 37 °C on LB medium with 1.5% agar for storage or without agar (liquid medium) for experiments.

### 2.4. Determination of the Toxicity of Silver Nanoclusters to Bacteria

Bacterial cultures were grown in liquid LB nutrient medium at 37 °C for 18 h (120 rpm). 25–50 µL of the overnight culture were added to a flask with 25 mL of sterile liquid LB medium and incubated under the same conditions for 1 h. The resulting bacterial suspension was used as an inoculum, 150 µL of which were added to the wells of a plate with 150 µL of a medium containing GSH-AgNCs in the concentration range from 0.002 to 0.25 mM (for silver). Wells with 150 µL of inoculant and 150 µL of LB medium were used as a control. Each experimental variant was performed in triplicate. Incubated at 37 °C 400 rpm for 18 h. Bacterial culture growth was assessed after 6 and 18 h by measuring optical density at 595 nm using a Multiskan Ascent plate reader (Thermo Labsystems, Helsinki, Finland).

### 2.5. Evaluation of the Effect of Silver Nanoclusters on Bacterial Resistance to Toxicants

Bacterial cultures were grown in liquid LB medium at 37 °C for 18 h (120 rpm). 25–50 µL of the overnight culture were added to a flask containing 25 mL of sterile liquid LB medium and incubated under the same conditions for 1 h. The resulting bacterial suspension was used as an inoculum, 150 µL of which were added to the wells of a plate with 150 µL of a medium containing heavy metal salts (concentrations, from 2.44 μM to 5 mM) and antibiotics (concentrations, from 5 ng/mL to 10 μg/mL). Afterwards, 1 µL of GSH-AgNCs solution was added to each well (the final concentration of nanoclusters (as silver) in the wells was 2.5 μg/mL). Incubated at 37 °C 400 rpm for 18 h. Each experiment was performed in triplicate. Bacterial culture growth was assessed by measuring optical density at 595 nm using a Multiskan Ascent plate reader (Thermo Labsystems, Helsinki, Finland).

The medium with toxicants was prepared as follows. 150 μL of LB medium were added to a row of wells of the plate. An additional 150 μL of medium containing 10 mM heavy metal salts (copper (II) chloride, cadmium (II) chloride) or 20 μg/mL antibiotics (erythromycin, levofloxacin) were added to the first well. The choice of toxicants was due to the fact that bacterial resistance to them is due to the functioning of RND efflux pumps containing TolC proteins [24,30]. The toxicological metrics of minimal inhibitory concentration (MIC), half maximal effective concentration (EC_50_), and maximal tolerable concentration (MTC) were determined by a graphical method based on the dependence of the optical density at 595 nm on the toxicant concentration.

### 2.6. Determination of the Concentration of Silver Nanoclusters Affecting Bacterial Resistance

To evaluate the effect of GSH-AgNCs doses on bacterial resistance to toxicants, nanocluster solutions were used at a concentration close to the MTC (2.5 μg/mL for silver), as well as at 10- and 100-fold lower concentrations. For this purpose, plates whose wells contained a series of two-fold dilutions of heavy metal salts and antibiotics in LB medium were prepared as described above. One hundred and fifty microliters of the inoculum were added to 150 μL of the medium containing toxicants. Then, 1 μL of GSH-AgNCs was added to separate series of toxicant dilutions to final concentrations of 0.025, 0.250, and 2.50 μg/mL (for silver). Wells without the addition of nanoclusters served as a control. Incubated at 37 °C 400 rpm for 18 h. Each experimental variant was performed in triplicate. Bacterial culture growth was assessed by measuring optical density at 595 nm using a Multiskan Ascent plate reader (Thermo Labsystems, Helsinki, Finland). The resistance of bacteria to toxicants was defined as the ability to grow (MIC value) at a concentration in the medium of heavy metals above 1 mM and antibiotics above 1 μg/mL.

## 3. Results

### 3.1. Preparation and Characterization of Silver Nanoclusters

**Figure 2:**
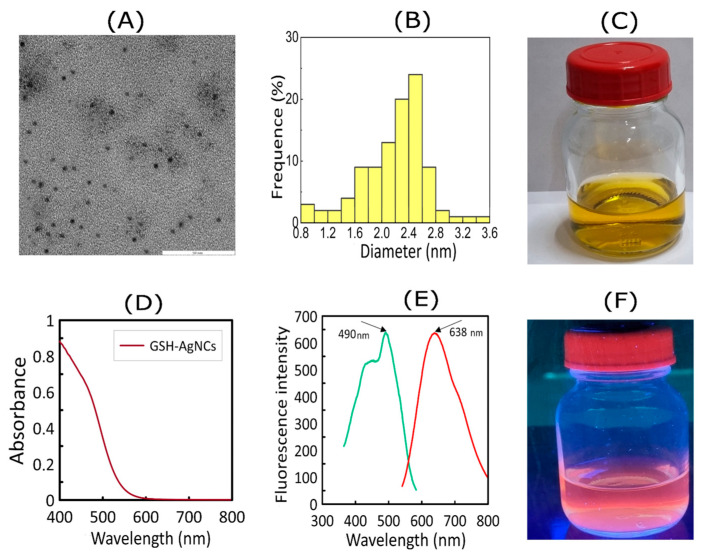
(**A**) Transmission electron microscope image of GSH-AgNCs. (**B**) Histogram showing the size distribution of the nanoclusters. (**C**) Photograph of the GSH-AgNCs suspension under daylight. (**D**) Extinction spectrum of GSH-AgNCs. (**E**) Fluorescence spectra of GSH-AgNCs. (**F**) Photograph of the GSH-AgNCs suspension under ultraviolet light.

Figure 2 shows TEM images, fluorescence and extinction spectra, and the appearance of the GSH-AgNCs colloidal solution.

As follows from the TEM data, the GSH-AgNCs samples are characterized by low polydispersity (Figure 2A). The average size of the synthesized GSH-AgNCs was 2.2 ± 0.5 nm (Figure 2B). These data are in good agreement with the standard size values of fluorescent metal nanoclusters, which were recorded in other studies [31]. Large (more than 5 nm) silver nanoparticles were absent in the obtained samples, which is further confirmed by the absence of a plasmon resonance peak in the extinction spectrum of GSH-AgNCs (Figure 2D). When illuminated with ultraviolet light, GSH-AgNCs fluoresced with red light (Figure 2F). Figure 2E shows the excitation and emission spectra of the synthesized nanoclusters. The recorded excitation and emission maxima were λ = 490 nm and λ = 638 nm, respectively. Thus, the Stokes shift of GSH-AgNCs is 148 nm, which is quite significant for silver nanoclusters. However, the synthesized nanoclusters are inferior in Stokes shift to typical fluorescent gold nanoclusters, for which the analogous indicator can be 200–300 nm [32]. It should be noted that the obtained GSH-AgNCs retained their fluorescent characteristics over a long period of storage, which indicates a high packing density of ligands–glutathione residues, which perform an additional protective function, preventing rapid oxidation of the silver core of the nanocluster by atmospheric oxygen.

**Figure 3:**
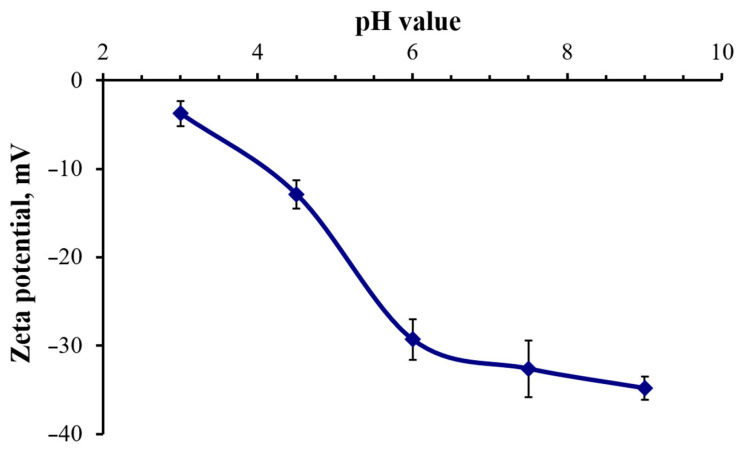
Changes in the zeta potential of GSH-AgNCs at different pH values. Bars show standard deviation.

Figure 3 shows the results of studying the synthesized nanoclusters using the Zetasizer Nano nanoparticle characterization system. It can be seen that the zeta potential of GSH-AgNCs at pH values of 6.0 and above is −30 mV, indicating a stable nanocluster solution. The negative zeta potential is due to the presence of deprotonated carboxyl groups of GSH. In an acidic medium (pH < 5.0), the carboxyl groups of GSH gradually protonate, leading to a decrease in the zeta potential of the nanoclusters and a reduction in the stability of their solution. In further experiments, the nanoclusters were added to LB nutrient medium, which has a pH value of approximately 7.2.

**Figure 4:**
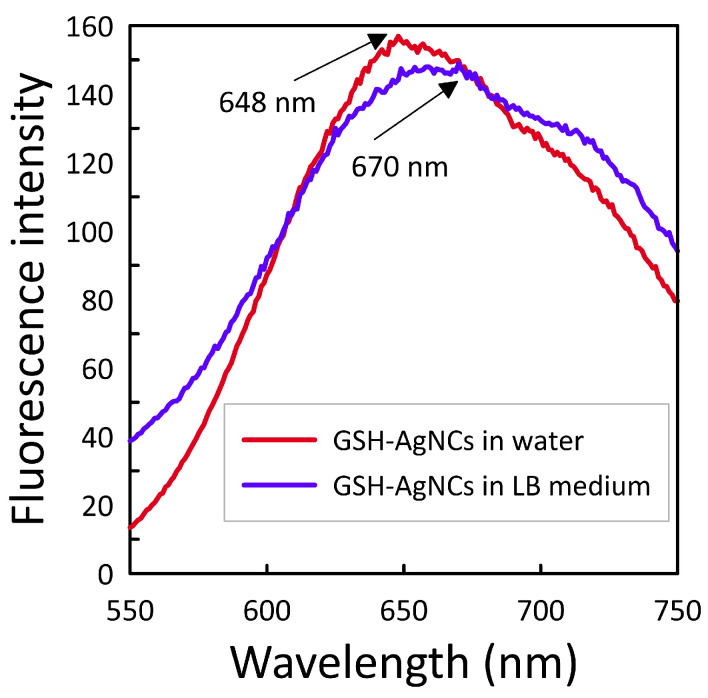
Fluorescence spectra of GSH-AgNCs recorded in different dispersion media: water and liquid LB culture medium.

The stability of GSH-AgNCs in LB culture medium was also assessed. We found that the GSH-AgNCs retained their fluorescent properties in LB medium, with a signal intensity virtually unchanged from the initial GSH-AgNCs (Figure 4). However, a red shift of approximately 20 nm was observed in the fluorescence emission spectrum. This shift is presumably due to interactions between the GSH-AgNCs and the peptides or peptones present in the culture medium. Fluorescent properties are a recognized marker of structural integrity. As no significant changes in fluorescent properties were noted, we conclude that GSH-AgNCs maintain high stability in LB medium.

### 3.2. Evaluation of the Antimicrobial Activity of Silver Nanoclusters

**Figure 5:**
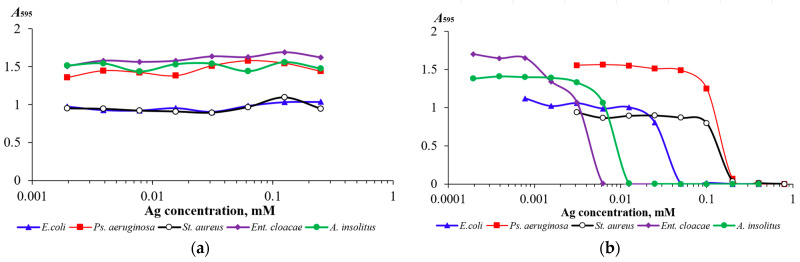
Dependences of the change in optical density of 18 h bacterial cultures of *Escherichia coli* K12, *Staphylococcus aureus* ATCC 25923, *Pseudomonas aeruginosa* ATCC 9027, *Achromobacter insolitus* LCu2, and *Enterobacter cloacae* K7 strains on the concentration of silver in the form of nanoclusters (**a**) and Ag^+^ cations (**b**) in the medium.

The resulting silver nanoclusters were tested for toxicity against five bacterial strains (*Escherichia coli* K12, *Staphylococcus aureus* ATCC 25923, *Pseudomonas aeruginosa* ATCC 9027, *Achromobacter insolitus* LCu2, and *Enterobacter cloacae* K7) by inhibiting culture growth in liquid LB medium (Figure 5a). The bacterial strains studied were found to have approximately equal resistance to GSH-AgNCs: dose-dependent growth inhibition was observed upon addition of nanoclusters containing 0.25 mM silver (1:10 of the initial GSH-AgNCs solution) to the nutrient medium only during the first hours of cultivation. Thus, for 6 h cultures, approximately 20% growth inhibition was detected in variants with the maximum concentration of GSH-AgNCs used in the experiment (Figure S1). We also tested the effect of 2.5 mM GSH, the same concentration used to synthesize the nanoclusters, on bacterial culture growth. Glutathione, an antioxidant and cellular metabolite, had no effect on bacterial growth at the concentration tested. Silver cations, on the other hand, inhibited bacterial growth significantly more than nanoclusters, based on the silver they contained (Figure 5b). Thus, GSH-AgNCs exhibit significantly less antimicrobial activity than silver(I) cations.

It should be noted that the studied strains differed significantly in their resistance to silver cations: *Staphylococcus aureus* ATCC 25923 and *Pseudomonas aeruginosa* ATCC 9027 were relatively resistant (MIC > 0.1 mM), while *Achromobacter insolitus* LCu2 and *Enterobacter cloacae* K7 were highly sensitive to silver cations (MIC ≈ 0.01 mM). GSH-AgNCs did not inhibit the growth of either the relatively resistant or the highly Ag^+^-sensitive bacterial strains.

### 3.3. The Effect of Silver Nanoclusters on Bacterial Resistance to Heavy Metals and Antibiotics

In the work of Tumskiy et al. (2023) [23], the interaction of GSH-AgNCs with the TolC and CusF proteins, which are involved in the removal of toxins from bacterial cells, was predicted. In this study, we assessed the effect of silver nanoclusters on bacterial resistance to heavy metals and antibiotics.

**Figure 6:**
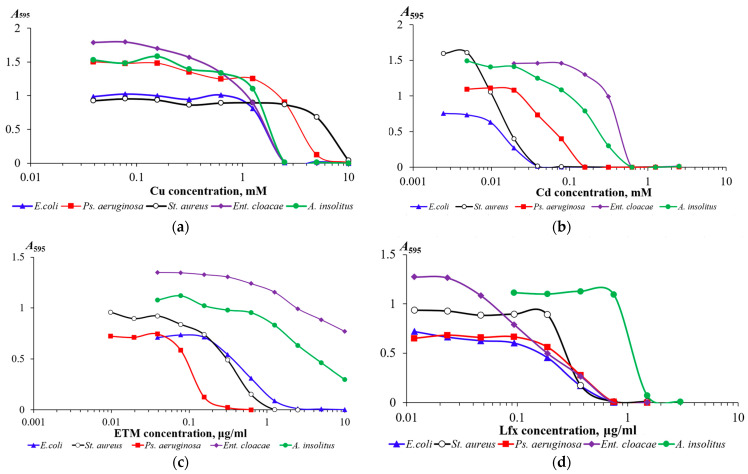
Dependences of the change in optical density of 18 h bacterial cultures of *Escherichia coli* K12, *Staphylococcus aureus* ATCC 25923, *Pseudomonas aeruginosa* ATCC 9027, *Achromobacter insolitus* LCu2, and *Enterobacter cloacae* K7 strains on the concentration of copper (**a**), cadmium (**b**), erythromycin (**c**), and levofloxacin (**d**) in the medium.

In the first stage, the resistance of bacterial strains to heavy metal salts (copper chloride (CuCl_2_ × 2H_2_O) and cadmium chloride (CdCl_2_ × H_2_O)), as well as to the antibiotics (erythromycin and levofloxacin), was determined (Table 1). The choice of toxins was determined because one of the mechanisms of bacterial resistance for these compounds is the functioning of RND efflux pumps, of which the TolC protein is a component. The studied strains demonstrated high resistance to copper (II) cations (MIC > 1.0 mM) (Figure 6a). The resistance of the strains to other toxicants varied. *Pseudomonas aeruginosa* ATCC 9027, *Achromobacter insolitus* LCu2, and *Enterobacter cloacae* K7 strains showed moderate resistance to cadmium cations (Figure 6b). All strains except *Pseudomonas aeruginosa* ATCC 9027 were resistant to erythromycin (Figure 6c). Conversely, only *Achromobacter insolitus* LCu2 was resistant to levofloxacin; the remaining strains showed moderate resistance to this antibiotic (1.0 μg/mL > MIC > 0.1 μg/mL) (Figure 6d).

**Figure 7:**
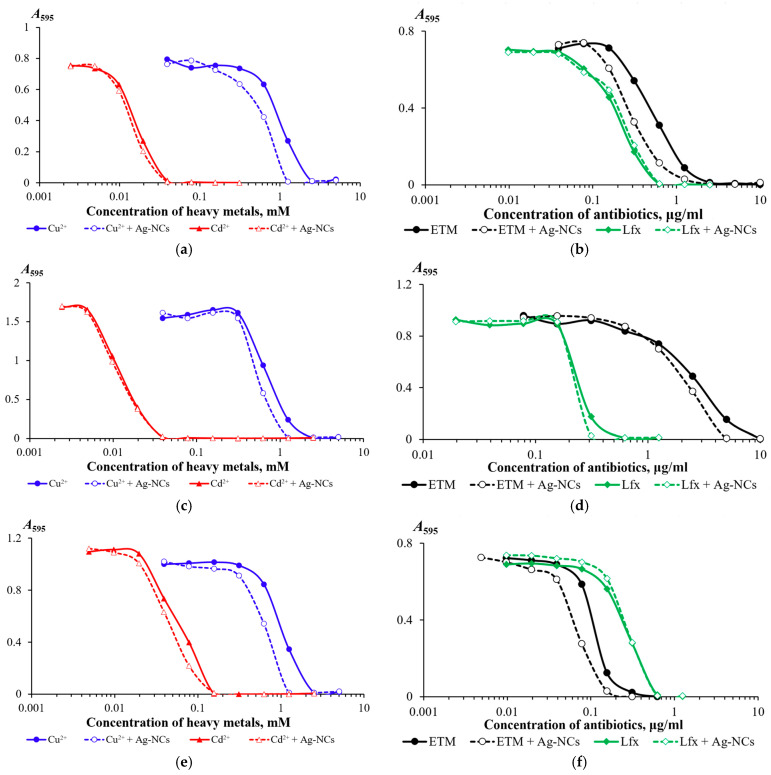

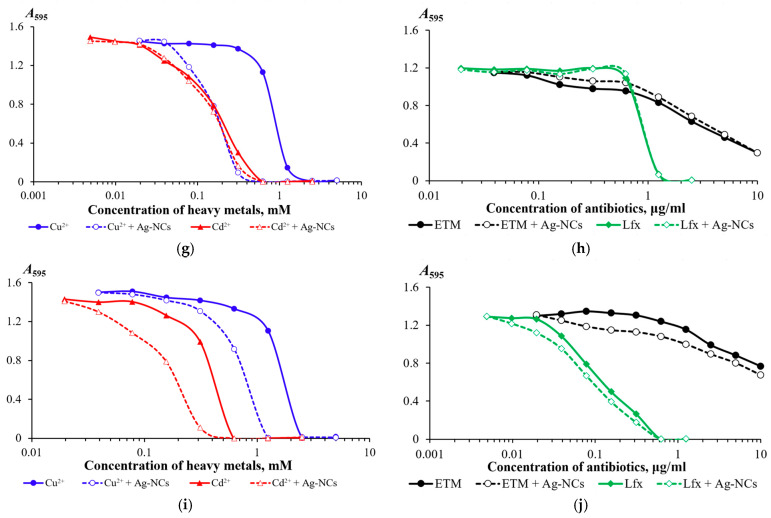
Dependences of the change in optical density of 18 h bacterial cultures of the strains *Escherichia coli* K12 (**a**,**b**), *Staphylococcus aureus* ATCC 25923 (**c**,**d**), *Pseudomonas aeruginosa* ATCC 9027 (**e**,**f**), *Achromobacter insolitus* LCu2 (**g**,**h**), and *Enterobacter cloacae* K7 (**i**,**j**) on the concentration in the medium of copper (Cu^2+^), cadmium (Cd^2+^) cations, erythromycin (ETM), and levofloxacin (Lfx) in the absence and addition of silver nanoclusters (GSH-AgNCs) to the medium.

Adding a 1:100 solution of silver nanoclusters to the bacterial culture medium (Figure 7) decreased toxicological metrics (MIC, EC_50_, and MTC), indicating a negative impact of silver nanoclusters on the functioning of bacterial defense mechanisms against toxicants (heavy metals and antibiotics). For all studied strains, adding AgNCs to the nutrient medium reduced copper resistance, with the greatest impact observed for the *Achromobacter insolitus* LCu2 strain (Figure 7g). A significant effect of GSH-AgNCs on cadmium resistance was detected only for the *Enterobacter cloacae* K7 strain (Figure 7i). Erythromycin resistance in the presence of AgNCs was slightly reduced for all strains studied. For *Staphylococcus aureus* ATCC 25923 and *Pseudomonas aeruginosa* ATCC 9027, the effect of AgNCs was manifested by a twofold decrease in MIC values (Figure 7d,f). For *Staphylococcus aureus* ATCC 25923, an effect of AgNCs on levofloxacin resistance was detected: a twofold decrease in MIC values (Figure 7d).

### 3.4. Effect of Different Concentrations of Silver Nanoclusters on Bacterial Resistance

**Figure 8:**
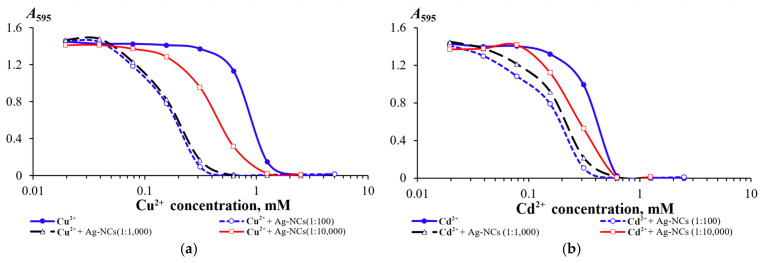

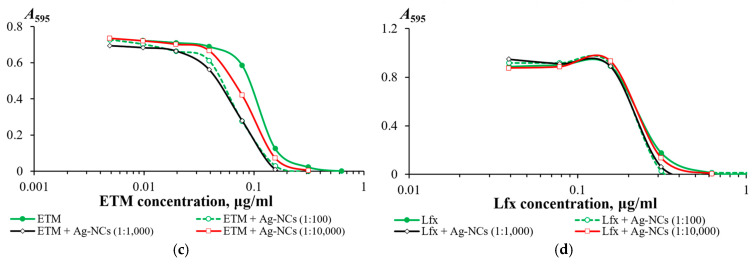
Dependences of the change in optical density of 18 h bacterial cultures of *Achromobacter insolitus* LCu2 strains on the concentration of copper cations in the medium (**a**), *Enterobacter cloacae* K7 on the concentration of cadmium cations (**b**), *Pseudomonas aeruginosa* ATCC 9027 on the concentration of erythromycin (**c**), and *Staphylococcus aureus* ATCC 25923 on the concentration of levofloxacin (**d**) upon the addition of GSH-AgNCs to the medium at concentrations of 2.5 (1:100), 0.25 (1:1000), and 0.025 (1:10,000) μg/mL (as silver).

For the toxicant-bacteria pairs (Cu^2+^—*Achromobacter insolitus* LCu2; Cd^2+^—*Enterobacter cloacae* K7; ETM—*Pseudomonas aeruginosa* ATCC 9027; and Lfx—*Staphylococcus aureus* ATCC 25923), for which the greatest effect of GSH-AgNCs was revealed, the change in bacterial resistance was studied upon the introduction of nanoclusters into the cultivation medium in ratios of 1:100; 1:1000; and 1:10,000) (Figure 8). It was found that a decrease in the concentration of nanoclusters below a ratio of 1:1000 leads to a rapid decrease in the effect on bacterial resistance to toxicants. Thus, a dose-dependent effect on the manifestation of bacterial resistance to heavy metal cations and antibiotics was revealed.

## 4. Discussion

Nanoparticles are widely used in biological and medical research. Some nanoparticles have been shown to exhibit antimicrobial activity against bacterial cultures and biofilms. Nanoclusters (due to their higher penetrating ability) are considered promising agents for combating bacteria. However, the antimicrobial activity of nanoclusters is not always detected. In this study, GSH-AgNCs were synthesized, for which an average diameter of 2.2 nm was determined using TEM and DLS. Fluorescence spectroscopy was used to determine the excitation (λ = 490 nm) and emission (λ = 638 nm) maxima (Figure 2). In LB medium, the GSH-AgNCs had the stability and retained their fluorescent properties, with a signal intensity virtually unchanged from the GSH-AgNCs in water (Figure 4). After washing the reaction mixture to remove reagents, the silver concentration in the GSH-AgNCs solution was 0.25 mg/mL, which is 2.3 mM. The characteristics of the obtained nanoclusters corresponded to those described previously [23] for AgNCs, for which interaction with bacterial TolC proteins was predicted.

The resulting GSH-AgNCs did not exhibit antimicrobial activity against the five bacterial strains studied: *Escherichia coli* K12, *Staphylococcus aureus* ATCC 25923, *Pseudomonas aeruginosa* ATCC 9027, *Achromobacter insolitus* LCu2, and *Enterobacter cloacae* K7. The observed slight inhibition of culture growth when nanoclusters were added to the medium at a ratio of 1:10 may be due to dilution of the bacterial culture medium. A comparison of the toxicity of GSH-AgNCs and AgNO_3_ towards the five bacterial strains studied (Figure 5) suggests the high stability of the GSH-AgNCs obtained in bacterial culture. When GSH-AgNCs were added to the culture medium of highly Ag^+^-sensitive strains (*Achromobacter insolitus* LCu2 and *Enterobacter cloacae* K7) at concentrations up to 25 μg/mL, no significant growth inhibition was observed. This indicates that the release of Ag^+^ from GSH-AgNCs (if it occurs) is so insignificant that it does not inhibit the growth of bacterial strains highly sensitive to silver.

We found that silver nanoclusters increase the sensitivity of bacteria to biocides such as heavy metal salts and antibiotics. Copper and cadmium chlorides were used in our study. Although we found no differences in toxicity for copper salts (chloride, sulfate, and acetate) towards the studied bacterial strains (unpublished data), the possible influence of salt anions on both the manifestation of heavy metal toxicity and the biological activity of GSH-AgNCs cannot be ruled out. GSH-AgNCs had the strongest negative effect on copper resistance of all the bacterial strains we studied, especially for *Achromobacter insolitus* LCu2 (Figure 7g) and *Enterobacter cloacae* K7 (Figure 7i), for which it was shown that, under the combined action of GSH-AgNCs and copper cations, bacterial growth was completely inhibited at copper cation concentrations at which bacterial growth without GSH-AgNCs did not differ from the control. GSH-AgNCs have previously been predicted to interact with the CusF protein [23], which is a periplasmic chaperone required for full resistance to copper [33]. Although we observed a decrease in the MIC of toxicants in the presence of GSH-AgNCs in accordance with the docking results [23], nanoclusters, due to their small size, can also penetrate the periplasm and cytoplasm of bacterial cells and are potentially capable of interacting not only with surface but also intracellular proteins [10,11], affecting the metabolism and viability of bacteria.

The reduction in bacterial resistance to erythromycin (and, to a lesser extent, to levofloxacin) that we observed (Figure 7) may presumably be due to the fact that nanoclusters bind to components of the outer membranes of RND efflux pump systems, such as channels formed by the proteins TolC, OprM, OprZ, and others. To date, various inhibitors of RND efflux pump systems of plant and microbial origin (reserpine and resveratrol [30,34,35]), as well as synthetic compounds (phenylalanyl arginyl β-naphthylamide (PAβN) [36], 1-(1-napthylmethyl)-piperazine (NMP) [37], carbonyl cyanide *m*-chlorophenylhydrazine (CCCP) [38], pyridoquinolone [39], and presumably some others [30,40]), have been described. These compounds are used to study the effect of the functioning of RND efflux systems on antimicrobial drugs against bacteria [41], including in the form of biofilms [42]. The effect of GSH-AgNCs on bacterial resistance to toxicants demonstrated in our work suggests that nanoclusters will be able to expand the set of tools for disrupting the functioning of microorganisms’ defense mechanisms.

In the future, it will be necessary to elucidate the reasons for the different effects of AgNCs on resistance to various toxicants in different bacteria. For example, why did GSH-AgNCs increase sensitivity to copper cations in all the bacteria we studied, but to cadmium cations in only one strain (*Enterobacter cloacae* K7)? Presumably, the differences in the effects of nanoclusters on bacterial resistance to copper and cadmium may indicate the functioning of at least two defense systems against these heavy metals: one sensitive and one insensitive to GSH-AgNCs. To maintain copper homeostasis, bacteria use several strategies: extracellular sequestration by biopolymers, binding by metallothionein proteins in the cytoplasm, and active release of the metal from the cell [43]. Genetic determinants of active copper transport from the cell include the *cop* and *cus* gene clusters. The CopA protein (P-type ATPase) transports Cu^+^ from the cytoplasm to the periplasm, where CueO (oxidase) oxidizes Cu^+^ to Cu^2+^, and the CusCFBA efflux system exports Cu^2+^ to the outside [26,44]. One of the active mechanisms of bacterial resistance to cadmium is also the functioning of the efflux pumps identified in the Czc or Cad systems [45]. Cus and Czc proteins may have different affinities for GSH-AgNCs. In the *Enterobacter cloacae* K7, either both systems are inhibited by GSH-AgNCs, or resistance to copper and cadmium is mediated by the functioning of a single RND efflux system.

Another important question for further research is to determine the limit of reduction in bacterial resistance to biocides under the influence of GSH-AgNCs. Notably, in our study, the copper resistance of the *Achromobacter insolitus* LCu2 strain, when exposed to AgNCs, was reduced to the level of sensitivity to cadmium, resistance to which was independent of the action of nanoclusters (Figure 7g). Based on this, it can be hypothesized that GSH-AgNCs reduce bacterial resistance to some toxicants from high to moderate. Also, an important direction for further development of this topic will be the experimental determination of GSH-AgNCs binding to RND efflux pump proteins and the determination of nanocluster saturation concentrations in the efflux systems of bacteria.

## 5. Conclusions

This study demonstrated that silver nanoclusters, while not exhibiting pronounced antimicrobial properties, enhance the toxic effects of certain heavy metals and antibiotics on bacteria. These results open up prospects for using silver nanoclusters as inhibitors of bacterial defense against drugs, which could be used, on the one hand, in research on new antibiotics, and on the other, as components that enhance the main active ingredient in the treatment of infections. However, this requires further study of the mechanism of action of nanoclusters on bacterial cells and their toxicity to animals and humans. In any case, the properties of nanoclusters described in this study may be useful in the search for ways to overcome multidrug resistance in bacteria.

## Figures and Tables

**Table 1:** Toxicological metric values—minimal inhibitory concentration (MIC) and half maximal effective concentration (EC_50_)—of heavy metal cations (copper and cadmium) and antibiotics (erythromycin and levofloxacin) for bacterial strains *Escherichia coli* K12, *Staphylococcus aureus* ATCC 25923, *Pseudomonas aeruginosa* ATCC 9027, *Achromobacter insolitus* LCu2, and *Enterobacter cloacae* K7.

Strains	Metrics	Cu^2+^, mM	Cd^2+^, mM	Erythromycin, μg/mL	Levofloxacin, μg/mL
*Escherichia coli* K12	MIC	3.0	0.08	2.5	0.7
	EC_50_	0.8	n.d. *	0.36	0.25
*Staphylococcus aureus* ATCC 25923	MIC	2.5	0.08	1.5	0.7
	EC_50_	0.75	n.d.	0.6	0.35
*Pseudomonas aeruginosa* ATCC 9027	MIC	3.0	0.2	0.6	0.7
	EC_50_	1.0	0.07	0.15	0.32
*Achromobacter insolitus* LCu2	MIC	2.0	0.61	>10	2.0
	EC_50_	0.8	0.18	7	1.1
*Enterobacter cloacae* K7	MIC	3.0	0.76	>10	0.8
	EC_50_	1.75	0.39	8	0.13

* not determined.

## Data Availability

Data are contained within the article and its Supplementary Materials. Additional data supporting the findings of this study are available from the corresponding author upon reasonable request.

## Supplementary Materials

The following supporting information can be downloaded at: https://www.mdpi.com/article/10.3390/nano16010054/s1, Figure S1: Dependences of the change in optical density of 6 h bacterial cultures of *Escherichia coli* K12, *Staphylococcus aureus* ATCC 25923, *Pseudomonas aeruginosa* ATCC 9027, *Achromobacter insolitus* LCu2, and *Enterobacter cloacae* K7 strains on the concentration of silver nanoclusters (in terms of the silver they contain).

## Abbreviations

The following abbreviations are used in this manuscript:

AgNCs	Silver nanoclusters
DLS	Dynamic light scattering
EC_50_	Half maximal effective concentration
ETM	Erythromycin
GSH	Glutathione
Lfx	Levofloxacin
MIC	Minimal inhibitory concentration
MTC	Minimal tolerable concentration
NPs	Nanoparticles
RND	“Resistance-nodulation-division”, the type of efflux pump systems
TEM	Transmission electron microscopy
LB	Lysogeny broth, rich nutrient medium
