# Supplementary material for: Silver Nanoclusters Decrease Bacterial Resistance to Heavy Metals and Antibiotics
Source: Nanomaterials (Basel). 2025 Dec 31;16(1):54. doi: 10.3390/nano16010054 (PMC12787750; doi:10.3390/nano16010054)
Supplement: Supplementary file 1 [file nanomaterials-16-00054-s001.zip › nanomaterials-4057672-supplementary.pdf]

nanomaterials

Supplemental Materials

Silver Nanoclusters Decrease Bacterial Resistance to Heavy Metals and Antibiotics

Gennady L. Burygin [1,2,*], Daniil S. Chumakov [1], Anastasia S. Astankova [1,2], Yulia A. Filip'echeva [1], Julia A. Balabanova [1] and Yelena V. Kryuchkova [1]

1 Institute of Biochemistry and Physiology of Plants and Microorganisms, Saratov Scientific Centre of the Russian Academy of Sciences, 13 Prospekt Entuziastov, 410049 Saratov, Russia; laik2012@yandex.ru (D.S.C.); asastankova@gmail.com (A.S.A.); ljuche@yandex.ru (Y.A.F.); yulia-kusmarceva@yandex.ru (J.A.B.); kryu-lena@yandex.ru (Y.V.K.)

2 Department Organic and Bioorganic Chemistry, Institute of Chemistry, Saratov State University, 83 Astrakhanskaya Street, 410012 Saratov, Russia

* Correspondence: buryginGL@gmail.com; Tel.: +7-8452-970383

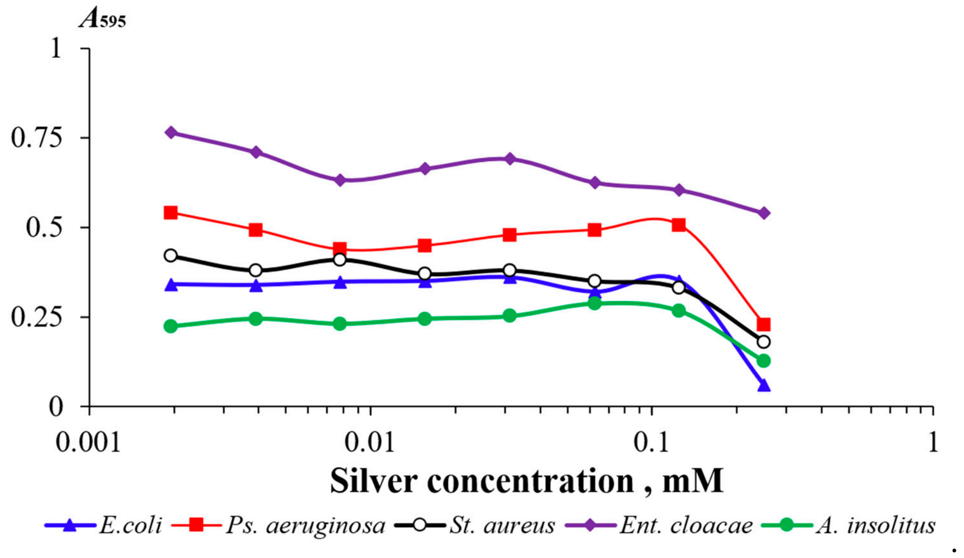

Figure S1. Dependences of the change in optical density of 6-hour bacterial cultures of *Escherichia coli* K12, *Staphylococcus aureus* ATCC 25923, *Pseudomonas aeruginosa* ATCC 9027, *Achromobacter insolitus* LCu2, and *Enterobacter cloacae* K7 strains on the concentration of silver nanoclusters (in terms of the silver they contain).

 https://doi.org/10.3390/nano16010054
